# Supplementary material for: Direct and Indirect Competitive Interactions between Ooencyrtus nezarae and Paratelenomus saccharalis Parasitizing Megacopta cribraria Egg Patches
Source: Insects. 2022 Dec 30;14(1):35. doi: 10.3390/insects14010035 (PMC9863137; doi:10.3390/insects14010035)
Supplement: Supplementary file 1 [file insects-14-00035-s001.zip › insects-2135449-supplementary.pdf]

Type of the Paper (Article)

Direct and indirect competitive interactions between *Ooencyrtus nezarae* and *Paratelenomus saccharalis* parasitizing *Megacopta cribraria* egg patches

Supplement file

Table S1. The proportion of unascribed eggs (unhatched or unparasitized) averaged across treatments in experiments conducted to examine how the order of *Ooencyrtus nezarae* and/or *Paratelenomus saccharalis* adult arrival at a *Megacopta cribraria* egg patch influences competition.

	**(Proportion± SE)**
**Treatment**	***M. cribraria* unascribed eggs**
O. nezarae* + *P. saccharalis	0.20 ± 0.024[b]
O. nezarae* → *P. saccharalis	0.40 ± 0.025[a]
P. saccharalis* → *O. nezarae	0.36 ± 0.025[a]
ANOVA for the main effect	F = 19.86; df = 2, 237; p = <0.0001

LS-means within a column followed by the same letter are not significantly different at $P < 0.05$. Data were presented with one-way ANOVA using simulated multiple comparison test.

Table S2. The proportion of unascribed eggs (unhatched or unparasitized) averaged across treatments in experiments conducted to examine how the order of *Ooencyrtus nezarae* and/or *Paratelenomus saccharalis* adult arrival at a *Megacopta cribraria* egg patch influences competition.

**(Proportion ± SE)**			
**Time interval**	***O. nezarae* + *P. saccharalis***	***O. nezarae* → *P. saccharalis***	***P. saccharalis* → *O. nezarae***
**12-h**	0.13 ± 0.04[d]	0.27 ± 0.04[cd]	0.33 ± 0.04[bcd]
**24-h**	0.16 ± 0.04[d]	0.21 ± 0.04[cd]	0.27 ± 0.04[cd]
**48-h**	0.20 ± 0.04[cd]	0.64 ± 0.04[a]	0.47 ± 0.04[ab]
**72-h**	0.29 ± 0.04[bcd]	0.47 ± 0.04[ab]	0.38 ± 0.04[bc]
**ANOVA table**			
**Treatment**	F = 26.40; df = 2, 228; p = <0.0001		
**time interval**	F = 19.57; df = 3, 228; p = <0.0001		
**Treatment x time interval**	F = 4.74; df = 6, 228; p = 0.0001		

LS-means within a column followed by the same letter are not significantly different at $P < 0.05$. Data were presented with two-way ANOVA using a simulated multiple comparison test.
